# CD14 Directs Adventitial Macrophage Precursor Recruitment: Role in Early Abdominal Aortic Aneurysm Formation

**DOI:** 10.1161/JAHA.112.000065

**Published:** 2013-04-24

**Authors:** Andra L. Blomkalns, Daniel Gavrila, Manesh Thomas, Bonnie S. Neltner, Victor M. Blanco, Stephanie B. Benjamin, Michael L. McCormick, Lynn L. Stoll, Gerene M. Denning, Sean P. Collins, Zhenyu Qin, Alan Daugherty, Lisa A. Cassis, Robert W. Thompson, Robert M. Weiss, Paul D. Lindower, Susan M. Pinney, Tapan Chatterjee, Neal L. Weintraub

**Affiliations:** 1 Division of Cardiovascular Diseases, University of Cincinnati, College of Medicine, Cincinnati, OH (B.S.N., Z.Q., T.C., N.L.W.); 2 Department of Emergency Medicine, University of Cincinnati, College of Medicine, Cincinnati, OH (A.L.B., V.M.B., S.B.B., S.P.C.); 3 Department of Environmental Health, University of Cincinnati, College of Medicine, Cincinnati, OH (S.M.P.); 4 Division of Cardiovascular Medicine, University of Iowa, Iowa City, Iowa (D.G., M.T., R.M.W., P.D.L.); 5 Department of Emergency Medicine, University of Iowa, Iowa City, Iowa (L.L.S., G.M.D.); 6 Department of Radiation Oncology, University of Iowa, Iowa City, Iowa (M.L.M.C.); 7 Department of Surgery (Section of Vascular Surgery), Washington University School of Medicine, St. Louis, Missouri (R.W.T.); 8 University of Kentucky, Lexington, Kentucky (A.D.); 9 Graduate Center for Nutritional Sciences, University of Kentucky, Lexington, Kentucky (L.A.C.)

**Keywords:** abdominal aortic aneurysm, CD14, innate immunity, macrophage, perivascular adipose tissue

## Abstract

**Background:**

Recruitment of macrophage precursors to the adventitia plays a key role in the pathogenesis of abdominal aortic aneurysms (AAAs), but molecular mechanisms remain undefined. The innate immune signaling molecule CD14 was reported to be upregulated in adventitial macrophages in a murine model of AAA and in monocytes cocultured with aortic adventitial fibroblasts (AoAf) in vitro*,* concurrent with increased interleukin‐6 (IL‐6) expression. We hypothesized that CD14 plays a crucial role in adventitial macrophage precursor recruitment early during AAA formation.

**Methods and Results:**

CD14^−/−^ mice were resistant to AAA formation induced by 2 different AAA induction models: aortic elastase infusion and systemic angiotensin II (AngII) infusion. *CD14* gene deletion led to reduced aortic macrophage infiltration and diminished elastin degradation. Adventitial monocyte binding to AngII‐infused aorta in vitro was dependent on CD14, and incubation of human acute monocytic leukemia cell line‐1 (THP‐1) monocytes with IL‐6 or conditioned medium from perivascular adipose tissue (PVAT) upregulated CD14 expression. Conditioned medium from AoAf and PVAT induced CD14‐dependent monocyte chemotaxis, which was potentiated by IL‐6. CD14 expression in aorta and plasma CD14 levels were increased in AAA patients compared with controls.

**Conclusions:**

These findings link CD14 innate immune signaling via a novel IL‐6 amplification loop to adventitial macrophage precursor recruitment in the pathogenesis of AAA.

## Introduction

Abdominal aortic aneurysm (AAA) disease is a frequent cause of morbidity and mortality, occurring in up to 12.5% and 5.2% of elderly men and women, respectively. Roughly 25 000 AAA repairs are performed each year, and despite the progress made in primary preventive measures and screening programs, AAAs account for >13 000 deaths annually in the United States.^1^ Only 21% to 33% of patients with aortic rupture survive to surgery, with an additional 50% mortality following surgery.^2^ Risk factors associated with AAA include older age, smoking, male sex, hypertension, and the presence of atherosclerotic disease in coronary or peripheral arteries.^3–4^ Despite the frequency and associated morbidity and mortality of AAA, the specific cellular mechanisms that underlie aneurysm formation and progression are poorly understood.

The immune system plays a key role in the pathogenesis of atherosclerosis and AAA. In the case of atherosclerosis, monocyte adhesion to inflamed endothelium is one of the earliest pathological features, followed by macrophage uptake of lipoproteins, leading to foam cell formation within the intima. The innate immune system, which is the genetically conserved, nonspecific first line of defense in the recognition of pathogens, has been linked to atherosclerosis in experimental animal models and in humans. For example, endotoxin (LPS) levels in the blood are strongly and independently associated with atherosclerosis in humans, and endotoxin injections augment atherosclerotic lesion development in animal models.^5–8^ Moreover, deletions of Toll‐like receptor 4 (TLR4) or its adaptor protein, MyD88, both of which play a crucial role in innate immune signaling, ameliorate atherosclerosis in mice.^9–10^

As in atherosclerosis, inflammation is central to AAA formation, but the specific role of the components of the innate immune system is less clear. Owens et al^11^ recently reported that deficiency of TLR4 or MyD88 reduced both angiotensin II (AngII)–induced atherosclerosis and AAA formation, indicating that innate immune signaling may contribute to the pathogenesis of AAA. In AAA, macrophages are recruited prominently to the adventitia and media, where they secrete proteases that lead to matrix degradation, smooth muscle cell apoptosis, tissue weakening, and aortic enlargement.^12–13^ Macrophage precursor recruitment to the adventitia during AngII‐induced AAA formation was reported to be dependent on adventitial secretion of interleukin‐6 (IL‐6) and monocyte chemotactic protein‐1 (MCP‐1) and of CCR2 expression in monocytes.^14^ The macrophages accumulating in aortic adventitia were CD14^hi^ and F4/80^−^, consistent with activated macrophages. Moreover, CD14 expression in monocytes in vitro was upregulated by coculture with fibroblasts, concurrent with increased secretion of IL‐6 and MCP‐1.

CD14 is a 55‐kDa GPI‐linked surface protein pattern recognition receptor that plays a central role in activation of the innate immune system through transduction of signals from bacterial LPS and various other ligands. CD14 is most known for its participation in signal transduction through TLR4 and, to a lesser extent, TLR2.^15–16^ Moreover, activated monocytes shed a soluble form of CD14 (sCD14) into the bloodstream, and levels of sCD14 have been shown to correlate with increased aortic stiffness in humans, further suggesting a role in vascular pathology.^17–19^

We hypothesized that CD14 plays a crucial role in macrophage precursor recruitment and thereby early aortic inflammation, leading to AAA formation. To test this hypothesis, we investigated the impact of *CD14* gene deletion on AAA formation and aortic pathology in 2 distinct murine models and in in vitro experiments with macrophage migration. To investigate our hypothesis in humans, we examined whether CD14 expression is locally enhanced in AAA and whether humans with AAA exhibit increased levels of sCD14 in plasma compared with age‐ and sex‐matched controls without AAA.

## Methods

### Elastase Infusion Murine AAA Model

Three‐ to 6‐month‐old (within 4 weeks of age for any 1 experimental set) C57Bl/6 (CD14^+/+^) and CD14^−/−^ on a C57Bl/6 background (Jackson) underwent the elastase model of aneurysm induction as previously described.^20^ All animals (CD14^+/+^, n=4; CD14^−/−^, n=5) were treated with elastase from the same lot. Heat‐inactivated (100°C×5 minutes) elastase served as the control (n=3). After 14 days, the aorta was reexposed, and final AD measurements were obtained. For individual animals, AAA was defined as an increase in diameter of ≥50% greater than the preperfusion diameter.

### AngII Infusion Murine AAA Model

For AngII experiments, apoE^−/−^/CD14^−/−^ mice were generated using apoE ^−/−^ CD14^+/−^ breeding pairs (Jackson). ApoE^−/−^/CD14^+/+^ littermates were used as controls. Three‐ to 6‐month‐old apoE^−/−^CD14^+/+^ and apoE^−/−^CD14^−/−^ mice (within 4 weeks of age for any 1 experimental set) were subjected to a 14‐day infusion of AngII (1000 ng/kg per minute) via subcutaneous osmotic minipumps (Model 2002, Durect Corporation) as described previously.^20^ Mice (CD14^+/+^, n=16; CD14^−/−^, n=7; saline‐infused controls, n=7) were euthanized after 14 days, and the abdominal aortas were exposed, measured in situ with digital calipers, and collected.

### Histological Studies

Formalin‐fixed, paraffin‐embedded tissue sections from mouse and human aortas were stained with hematoxylin and eosin and Verhoeff–van Gieson (VVG) stain (elastin). Primary antibodies included rabbit anti‐mouse CD14 (Santa Cruz M‐305; 1:100); mouse anti‐human CD68 (Dako; Clone PG‐M1; 1:100); rat anti‐mouse Mac‐3 antibody (BD Pharmingen; 1:100); rabbit anti‐human CD14 (Epitomics; 1:200). Species and isotype‐matched antibodies were used as controls in the mouse studies. In human studies, omission of the primary antibody served as the negative control.

### Zymographic Analysis of Matrix Metalloproteinase MMP‐2 and MMP‐9 Activity

MMP‐2 and MMP‐9 activity was determined by zymography as described previously.^21^ Briefly, prepoured 10% polyacrylamide gels containing 0.1% gelatin A were purchased (Bio‐Rad) and used as the substrate for MMP activity. Equivalent amounts of samples were loaded on the basis of protein content (Bradford assay; Bio‐Rad).

### Image Quantification

ImageJ software (version 1.42; National Institutes of Health) was used for quantitative analysis. Bands were quantified on the basis of their relative intensities. Each aorta image was scanned in 4 nonoverlapping fields, and average values are reported in the Table.

**Table 1.** Summary of Image Quantifications

Manuscript Reference	Measurement	Control	Genotype CD14^+/+^	Genotype CD14^−/−^	*P* Value
Elastase H and E (Figure 1B)	Average particles (PMNs)/field	13±3.2* (CD14^+/+^ HI elastase)	20±6.5	15±3.3	0.03
Elastase Mac‐3 (Figure 1B)	Average particles (macs)/field	4±1.25* (CD14^+/+^ HI elastase)	16±1.43	6±0.75	0.04
Elastase VVG (Figure 1B)	Elastin band area/field (μm^2^)	151 969±8134* (CD14^+/+^ HI elastase)	116 936±6841	142 873±27 497	<0.001
AngII H and E (Figure 2C)	Average particles (PMNs)/field	30±6.0		18±3.66	0.08
AngII Mac‐3 (Figure 2D)	Brown stain/field	11±1.4 (thoracic)		18±3.0 (abd)	0.04
Zymogram elastase model	MMP‐2, area%		7.65±0.38	5.31±0.77	0.04
Zymogram elastase model	MMP‐9, area%		4.60±0.32	2.80±0.61	0.04
Zymogram AngII model	MMP‐2, area%	9.26±0.41		8.60±0.78	0.49
Zymogram AngII model	MMP‐9, area%	3.91±0.40		3.24±0.87	0.25
		Condition or location			
IL‐6 migration (Figure 3C)	Blue fluorescence	39±3.4 (LAM only)	77±6.7 (LAM+IL‐6)	Single image	

All images from this set of experiments were quantified using the publically available NIH Image J/FIJI software. Four similar fields per sample were quantified and the values averaged according to standard instructions of Image J/FIJI densitometry software (“Madison,” National Institutes of Health, Bethesda, MD). Quantifications include number of cells, density, and percent area affected and are designated in the Measurement column. Controls include vehicle administration and/or wild‐type genotype and are designated in the labeled columns. Standard errors and statistics are provided where applicable. PMN indicates polymorphonuclear; VVG, Verhoeff–van Gieson stain; H and E, hematoxylin and eosin; AngII, angiotensin II; MMP, matrix metalloproteinase; IL, interleukin; AAA, abdominal aortic aneurysm; LAM, lipoarabinomannan.

* Heat‐inactivated (HI) elastase control in the elastase AAA model.

### Cell Isolation and Culture

All cell isolation and culture experiments were performed in triplicate. Human acute monocytic leukemia cell line‐1 (THP‐1) monocytic cells (ATCC) were cultured, and expression of CD14 was induced by calcium supplementation for 3 days. Mouse peritoneal macrophages were harvested 4 days after injection of 3% thioglycollate. Human aortic adventitial fibroblasts (AoAf) obtained from Lonza were grown in recommended medium. Conditioned medium was collected and stored at −80°C after culturing subconfluent cells.

To investigate the dependence of vascular monocyte binding on CD14, we incubated fluorescently labeled macrophage precursors with aorta vessel explants as previously described.^22^ ApoE^−/−^ mice were infused with AngII for 4 days, and aortas were removed, segmented, and incubated in vitro with fluorescently labeled CD14^+/+^ or CD14^−/−^ macrophages (200 000 cells in 100 μL of medium) for 2 hours. Embedded aortic segments were sectioned in 10‐μm sections. For every 2 aortic segments (ie, each well), macrophages bound to the adventitia and intima were counted and averaged.

### Cell Migration Experiments

THP‐1 cells and mouse peritoneal macrophages were used in this set of experiments. All experiments were performed in triplicate. LAM and MCP‐1 served as positive and negative controls, respectively, for CD14‐dependent responses.^23^ Migration to conditioned media from human AoAf and PVAT was studied. THP‐1 cells were incubated with isotypic IgG2 or anti‐CD14 (MY4; Beckman Coulter) monoclonal antibodies, and cell migration was assayed with chemotaxis chambers (Neuro Probe, Inc) in triplicate. Results are expressed as percent increase in migration relative to migration toward the control medium.

### Flow Cytometry

THP‐1 cells were used to measure levels of CD14 expression after incubation with varying concentrations of IL‐6, MCP‐1, osteopontin, or conditioned medium from PVAT. These experiments were performed in triplicate. Cells were incubated with human CD14‐FITC antibody (CD14, Mouse Anti‐Human, FITC, Invitrogen) to detect CD14 expression, and mouse IgG2a isotype (Mouse, IgG2a, FITC, Invitrogen) was used as a control. All samples were analyzed using CELL Quest Pro Software, and data are reported in compliance with MIFlowCyt standards.^24^

### Human AAA Specimens

Aneurysmal and adjacent nonaneurysmal human aorta samples were obtained during surgery from 3 patients undergoing AAA repair. Removal of these tissues was accomplished as part of the normal operative procedure, in which both aneurysm and adjacent nonaneurysmal segments were trimmed in preparation for graft anastomosis. These were initially fixed and paraffin‐embedded and then mounted in 5‐μm sections. CD14 was detected by a polyclonal goat anti‐mouse CD14 antibody (sc‐6999; Santa Cruz Biotechnology, Santa Cruz, CA) and then processed with a commercial immunoperoxidase staining kit (Vectastain Elite ABC Kit; Vector Labs). In these experiments, specimens incubated with no primary antibody served as negative controls.

### Measurement of Soluble CD14 in Human Serum

Human serum samples were obtained from the Fernald Medical Monitoring Program population cohort.^25–26^ The Fernald data are part of a longitudinal medical monitoring program that includes collection of routine serum samples. These patients are not part of a blinded, controlled trial but rather an observational cohort. We obtained human samples from this diverse population over a 20‐year period. In that these patients were not selected on the basis of any study‐related inclusion or exclusion criteria (except living in the Fernald affected area), 21 patients with AAA documented by imaging (CT scan or ultrasound) or surgical pathology were identified through medical record review. Controls (n=28) consisted of age‐ and sex‐matched patients without AAA (n=21) as documented by imaging studies. Soluble CD14 (sCD14) levels were determined using a CD14 ELISA assay kit (R&D Systems). The nonparametric Mann–Whitney *U* test was used to compare the sCD14 levels between cases and controls.

### Approvals

All protocols were approved by the Institutional Animal Care and Use Committees and Review Boards at the Universities of Iowa and Cincinnati.

### Statistical Analysis

Results are expressed as mean±SEM. Group differences were analyzed by the Student *t* test and multiple groups by 1‐way ANOVA. Fisher's exact test was used to analyze categorical data. *P*<0.05 was considered significant.

## Results

To determine the effects of *CD14* gene deletion on experimental aneurysm formation, we employed 2 widely accepted yet disparate murine models: (1) intra‐aortic infusion of elastase in CD14^+/+^ and CD14^−/−^ mice and (2) infusion of AngII via an osmotic minipump in hyperlipidemic apoE^−/−^ mice.^20^ We chose to examine aortic changes at an earlier point, 14 days, as opposed to the previously published and more common 28‐day experiments. Both models historically yield AAAs in >90% of mice and exhibit pathological evidence of inflammation and matrix degradation, typical features of human AAAs. Image quantification was performed on microscopic sections (Table).

### Deletion of *CD14* Gene Prevented AAA Formation in Elastase‐Infused Mice

**Figure 1.**
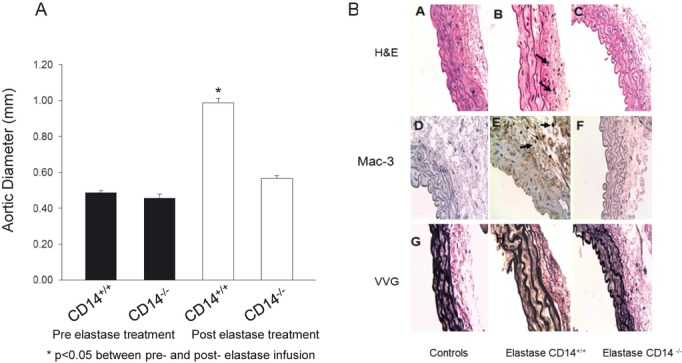
A, Deletion of CD14‐attenuated elastase‐induced AAA formation (CD14^+/+^, n=4; CD14^−/−^, n=5). B, Representative aortic histology demonstrating inflammatory cell infiltration (top), macrophages (middle), and elastin band staining (bottom). The luminal side of the vessel is to the right in the images. Heat‐inactivated elastase in CD14^+/+^ (n=3) was used to control for surgical manipulation. Image quantification revealed a significant difference in macrophage infiltration and elastin degradation between CD14^+/+^ and CD14^−/−^ animals (shown in Table). AAA indicates abdominal aortic aneurysm; H&E, hematoxylin and eosin; VVG, Verhoeff–van Gieson stain.

We sought to determine if *CD14* deletion would attenuate aneurysm formation in a murine AAA model that was independent of atherosclerosis, hypertension, and hyperlipidemia. Infusion of elastase induced an increase in final aortic diameter in CD14^+/+^ mice, consistent with AAA formation. In contrast, none of the CD14^−/−^ mice developed AAAs following elastase infusion (Figure 1A).

### *CD14* Deletion Attenuated Inflammatory Cell Infiltration

Examination of aortic histology by H&E staining (Figure 1B, A through C) showed marked inflammatory cell infiltration in the CD14^+/+^ mice infused with elastase, particularly in the adventitial region, which was attenuated in CD14^−/−^ mice. Many of these adventitial cells stained positively for MAC‐3, a macrophage‐specific antibody (Figure 1B, D through F) in elastase‐infused CD14^+/+^ mice, whereas minimal staining was observed in CD14^−/−^ mice. VVG staining (Figure 1B, G through I) demonstrated that elastin bands were largely preserved in control mice, which received heat‐inactivated elastase, and likewise in elastase‐infused CD14^−/−^ mice. In contrast, subtle elastin band flattening and straightening of the wavelike appearance were observed in elastase‐infused CD14^+/+^ mice. Also, MMP‐2 and MMP‐9 activity was slightly reduced in elastase‐infused CD14^−/−^ mice compared with CD14^+/+^ mice (Table).

### Deletion of *CD14* Gene Reduced Incidence and Severity of Aneurysms in AngII‐Infused ApoE^−/−^ Mice

**Figure 2.**
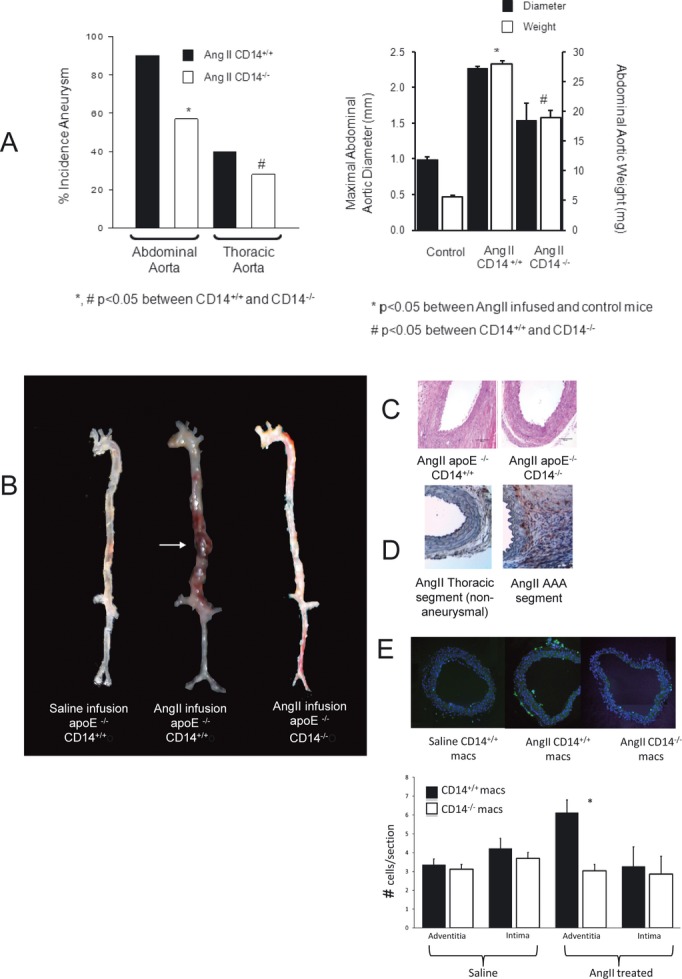
A, Deletion of CD14 reduced the incidence of AAA and TAA induced by AngII infusion (left). Effects of CD14 deletion on AAA diameter and weight (right). B, Representative gross images of aortas harvested from saline‐infused (control, n=7) and from AngII‐infused mice (CD14^+/+^, n=16; CD14^−/−^, n=7). C, Representative H&E staining and D, macrophage immunostaining (lower) in suprarenal and thoracic aortic segments.E, Representative images of CD14^+/+^ and CD14^−/−^ monocyte binding to aortic segments from saline‐ and AngII‐infused mice with accompanying bar graph of quantification. AAA indicates abdominal aortic aneurysm; TAA, thoracic aortic aneurysm; AngII, angiotensin II; H&E, hematoxylin and eosin.

In this set of experiments we sought to determine the effect of *CD14* deletion in a different model of AAA formation. Subcutaneous infusion of AngII for 2 weeks produced AAAs in 92% and thoracic aortic aneurysms (TAAs) in 42% of apoE^−/−^ mice, whereas no animals infused with saline developed AAA. Deletion of *CD14* reduced the incidence of AAAs and TAAs induced by AngII infusion as well as aneurysm diameter and weight (Figure 2A). Blood pressure measurements did not differ between the CD14^−/−^ animals and the CD14^+/+^ animals (Table). Representative pictures of aortas from these animals are shown in Figure 2B. Aneurysms in this model typically form in the suprarenal aorta in conjunction with a thrombus. In addition, AAA pathology (assessed using a standardized pathology scoring system that takes into account the number of discrete aneurysms and the presence of thrombus formation)^27^ was diminished in the CD14^−/−^ mice. MMP‐2/9 activity (Table) did not differ between the 2 groups.

### *CD14* Deletion Attenuated Inflammatory Cell Infiltration in AngII‐Infused apoE^−/−^ Mice

As macrophages are indispensable for AAA development and macrophage precursor infiltration may be an initial step in AAA formation in this model,^20,28^ we examined the extent of macrophage accumulation within aortic segments. In AngII‐infused apoE^−/−^ mice, aortas displayed evidence of increased wall thickness and inflammatory cell infiltration, particularly in the adventitial region, which was strongly abrogated in mice lacking CD14 (Figure 2C). Many of the inflammatory cells infiltrating the adventitia of AngII‐infused apoE^−/−^ mice stained positively for a macrophage marker (Figure 2D). Serum monocyte proportions of the total white blood cell count did not differ between CD14^+/+^ and CD14^−/−^ mice (31±12% and 45±10%, respectively, *P*=NS). Notably, macrophage infiltration was much less in nonaneurysmal thoracic aortic segments from these same mice. These results indicate that adventitial macrophage infiltration is colocalized to the site of AAA formation in this model.

Next, we investigated the role of CD14 in aortic macrophage precursor recruitment after only 4 days of AngII infusion, early during the course of AAA formation, using an in vitro assay.^22,29^ Avid binding of CD14^+/+^, but not CD14^−/−^, macrophage precursors to aortic explant adventitia was detected (Figure 2E) in comparison with the luminal surface. These results confirm that CD14 expression in monocytes and macrophage precursors is crucial for their recruitment to adventitia early during AngII‐infused AAA formation.

### IL‐6 Amplified CD14 Expression and CD14‐Dependent Monocyte Migration

**Figure 3.**
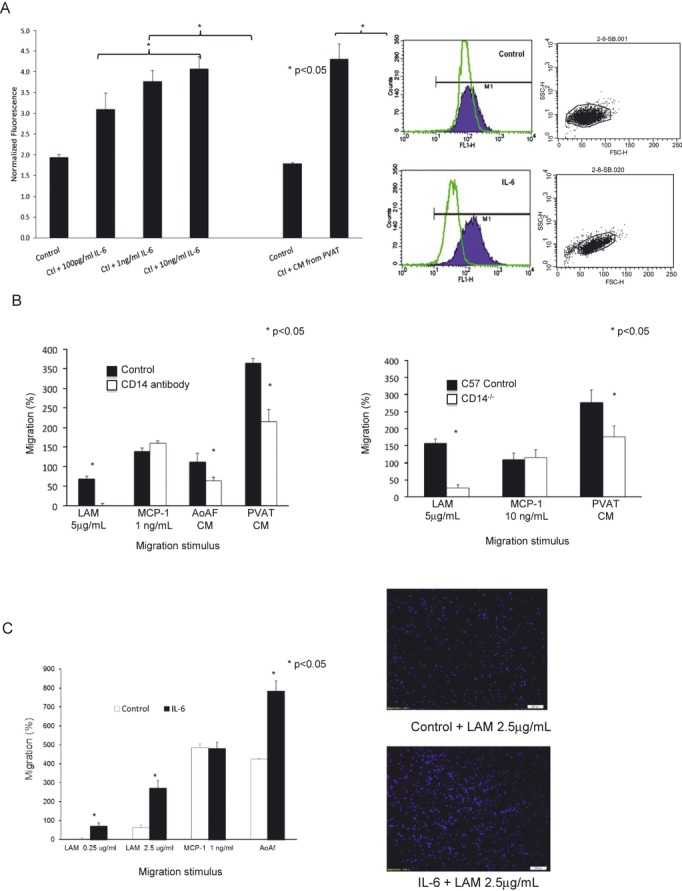
All experiments were performed in triplicate. A, Cumulative data (left), representative histogram (middle), and dot blots (right) showing effects of IL‐6‐ and PVAT‐conditioned media on CD14 expression in THP‐1 cells. The green outline peak represents the expected background staining, or “unstained control.” The filled‐in purple curves show the response of the isotype control (top) as the negative control or the IL‐6 (bottom). B, Migratory responses to LAM‐, MCP‐1‐, AoAf‐, and PVAT‐conditioned media in THP‐1 cells with and without CD14 blocking CD14 (left panel), and in peritoneal macrophages from CD14^+/+^ and CD14^−/−^ mice (right panel). Note that different concentrations of MCP‐1 were used in these experiments employing THP‐1 cells (left) or murine peritoneal macrophages (right). C, Cumulative data (left) and representative images showing effects of IL‐6 preincubation on migration of THP‐1 cells in response to 2 concentrations of LAM‐, MCP‐1‐, and AoAf‐conditioned media. IL indicates interleukin; LAM, lipoarabinomannan; MCP‐1, monocyte chemotactic protein; PVAT, perivascular adipose tissue; AoAf, aortic adventitial fibroblast; THP‐1, human acute monocytic leukemia cell line.

Prior studies have suggested that IL‐6, a key cytokine in AAA, upregulates CD14 expression as part of a feed‐forward inflammatory loop.^14^ We observed that IL‐6 dose‐dependently upregulated CD14 expression in human THP‐1 monocytic cells (Figure 3A). CD14 expression was likewise increased by exposure to conditioned medium from PVAT, which directly abuts the vascular adventitia and is a rich source of IL‐6.^30^ This suggests that PVAT could play a role in upregulating adventitial CD14 expression during AAA formation. In contrast, neither MCP‐1 nor osteopontin, 2 cytokines, which have likewise been implicated in AAA formation,^14,31^ affected CD14 expression in THP‐1 cells (data not shown).

THP‐1 chemotaxis was used to investigate the role of CD14 in human monocyte migration. Migration to conditioned media from human AoAf and PVAT was partially inhibited by the CD14‐blocking antibody (Figure 3B). Likewise, in murine peritoneal macrophages (Figure 3B, right), *CD14* gene deletion attenuated migration to LAM‐ and PVAT‐ conditioned media. Finally, preincubation with IL‐6 potentiated LAM‐induced migration, consistent with enhanced CD14‐dependent migratory responses. Moreover, migration induced by AoAf‐conditioned medium was potentiated by preincubation with IL‐6, whereas migration to MCP‐1 was unaffected (Figure 3C).

### CD14 Expression in Human AAA

**Figure 4.**
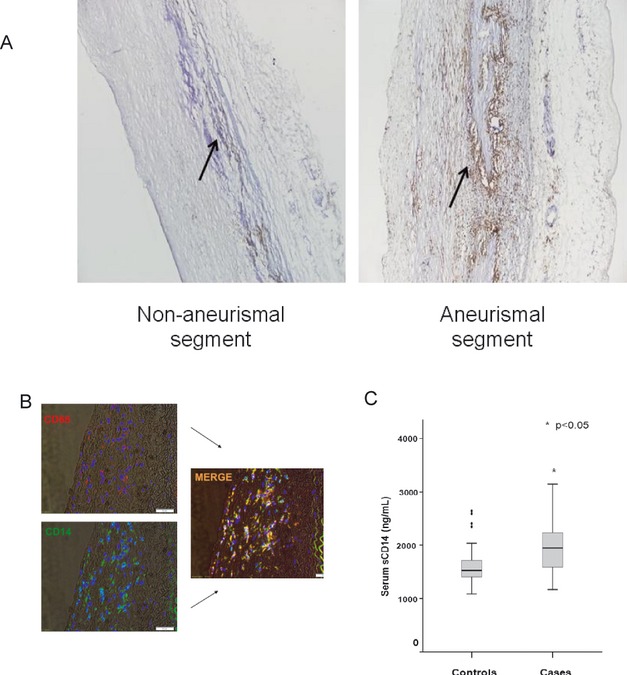
A, Representative images from 3 patients, expression of CD14 (arrows) in representative human AAA (right) and adjacent nonaneurysmal aorta (left) in the same patient. B, Immunofluourescence localizing macrophages (CD68, red) and CD14 (green) in human AAA tissue; nuclei are stained blue. A merged image appears on the bottom right of the fluorescent panels. C, sCD14 levels in sera from 28 age‐ and sex‐matched control patients and 21 patients with AAAs. Data are presented in a box‐and‐whisker diagram; •,outliers. AAA indicates abdominal aortic aneurysm; sCD14, soluble form of CD14.

We sought to validate whether CD14 expression is associated with human AAAs. First, we compared CD14 expression in segments of infrarenal AAAs and adjacent nonaneurysmal tissues obtained from patients (n=3) undergoing elective AAA repair. As expected, the tissue was severely degenerated, in keeping with the end‐stage nature of the disease. Examination of tissue histology showed atherosclerotic changes (ie, neointimal proliferation, foam cell formation) in both AAAs and nonaneurysmal sections of aorta, whereas inflammation, medial degeneration, and oxidative stress were prominent in the AAA tissues, as has been previously reported by Miller et al.^32^ Aortic tissue sections were immunostained for CD14, which showed increased expression in AAA compared with non‐aneurysmal aortic tissues from the same patient (Figure 4A); immunostaining was not detected in the negative controls without primary antibody (not shown). CD14 expression was increased throughout all layers of AAA and colocalized with macrophages (Figure 4B). Second, we sought to translate these observations to an available medical‐monitored cohort with banked serum samples. We were able to identify a group of patients with AAAs documented by aortic imaging and/or surgical pathology and compared serum sCD14 concentrations to age‐ and sex‐matched control patients in whom the absence of AAA was verified by aortic imaging. As shown in Figure 4C, although there was considerable variability, sCD14 concentrations were higher in AAA cases compared with controls (28 control patients and 21 patients with AAAs) when evaluated by the Mann–Whitney *U* test (*P*=0.02). Together, these data suggest that CD14 expression may be locally and systemically upregulated in human AAA.

## Discussion

The innate immune signaling molecule CD14 is both a marker for monocytes/macrophages and a transducer of immune responses. We report that CD14 is requisite for adventitial recruitment of macrophage precursors leading to aneurysm formation. To our knowledge, this is the first report demonstrating a confirmed role for CD14 in the pathogenesis of vascular disease.

The innate immune system is the first line of defense for invading microorganisms and has been shown to be involved in atherosclerotic disease^9,33^ TLRs are central to this process by recognizing pathogen‐associated molecular patterns (PAMPs), including bacterial endotoxin. Responses to LPS are enhanced by CD14, which, in cooperation with LPS‐binding protein, facilitates binding and transfer of endotoxin to the TLR4/MD‐2 complex.^34^ CD14 is expressed on several cell types including neutrophils, dendritic cells, lymphocytes, and even nonmyeloid cells, but at concentrations much less than on monocytes and macrophages.^35^ Mice deficient in CD14 exhibit reduced systemic inflammation and improved survival in response to injected endotoxin, indicating the importance of CD14 in endotoxin signaling in vivo.^36^ Combined inhibition of CD14 and complement dramatically reduced inflammation induced by *Escherichia coli* in pig whole blood, suggesting a promising strategy to treat gram‐negative sepsis.^37^ In vitro studies indicate that smooth LPS requires CD14 for activation of downstream inflammatory signaling through the TRIF/TRAM pathway. In contrast, the lipid A molecule (rough LPS) can activate TLR4 in the absence of CD14 through recruitment of MyD88 and Mal.^38^

The classic CD14 ligand is bacterial endotoxin, which has been suggested to play a role in AAA formation in humans and mice.^39^ However, other factors in the AAA milieu may also contribute to the disease process in a CD14‐dependent manner. For example, cell‐surface CD14 specifically binds minimally modified (oxidized) LDL, which plays an important role in vascular disease.^33,40^ CD14 has been postulated to play a role in delivering TLR ligands to lipid raft microdomains, thereby facilitating interactions with kinases and G‐proteins coupled to inflammatory signaling.^41–42^ Also, CD14 has been demonstrated to mediate monocyte‐induced T‐cell activation and monocyte binding to cytokine‐stimulated endothelial cells, suggesting that this molecule regulates many aspects of inflammation pertinent to cardiovascular diseases such as atherosclerosis and AAA.^43^ Although the findings of this study clearly implicate CD14 in the pathogenesis of AAA, identification of the specific CD14 ligands and downstream signaling pathways remains to be determined.

Several studies have suggested an important role for innate immunity in the formation of atherosclerotic disease.^44–45^ However, as in AAA, the specific PAMPs involved, receptors, coreceptors, and adaptor proteins that transduce the inflammatory signals, remain to be fully elucidated. In addition to bacterial products produced during indolent infections, modified host ligands, such as dying cells and oxidized lipid molecules present in the atherosclerotic milieu, likely serve as PAMPs in the setting of atherosclerosis.^46–47^ Consistent with this notion, TLR2, TLR4, and the TLR4 adaptor protein MyD88 have been implicated in the pathogenesis of atherosclerosis in murine models.^9,33,48^ Because CD14 interacts with both TLR4 and TLR2 and mediates cytokine‐induced monocyte adherence to endothelial cells, it would seem intuitive that CD14 should also be involved in atherosclerosis. However, Bjorkbacka et al^9^ showed that deletion of CD14 had no impact on aortic root atherosclerosis in apoE^−/−^ mice.

In contrast to the data suggesting a lack of involvement of CD14 in atherosclerosis, we provide compelling evidence that CD14 is intimately involved in AAA formation. We chose 2 complementary murine models in which to address our hypothesis: intra‐aortic elastase infusion and systemic AngII infusion in hyperlipidemic mice. As is the case with many small‐animal models of human disease, these models have unique strengths and weaknesses, and neither recapitulates all features of human AAA with complete fidelity. The elastase infusion model bears many similarities to human AAA, including inflammatory cell infiltration, MMP activation, and elastin degradation. However, rupture and thrombus formation are uncommon in this model. In contrast, rupture and thrombus formation occur frequently in the AngII infusion model, although the location tends to be medial and/or adventitial rather than luminal, which is perhaps more consistent with aortic dissection rather than AAA. In addition, AAA induced by AngII infusion is, like human AAA, more prevalent in men and positively influenced by hyperlipidemia.^20^ That mice lacking CD14 were protected against AAA formation induced by intra‐aortic elastase infusion and systemic AngII infusion suggests that CD14 is required for pathological processes common to both experimental models. Deletion of CD14 had little impact on activation of MMP‐2/9, key proteases involved in matrix degradation leading to aortic expansion. In contrast, adventitial macrophage precursor recruitment, a key initiating step in both models of AAA formation, was strongly abrogated in mice lacking CD14.

Very little is known about mechanisms that regulate adventitial inflammation in diseases such as AAA. Adventitial cells, including AoAf and perivascular adipocytes, are active participants in this process, secreting factors that contribute to matrix remodeling and inflammatory cell recruitment.^30^ Indeed, we previously reported that perivascular adipocytes secrete higher levels of proinflammatory cytokines than subcutaneous or visceral adipocytes.^30^ Here, we have provided novel insight into mechanistic interactions by demonstrating that factors secreted by adventitial cells upregulate monocyte expression of CD14, which in turn directs monocyte migration. We have further demonstrated that IL‐6 potently upregulates CD14 expression, whereas MCP‐1 and osteopontin, proinflammatory cytokines that are also implicated in AAA, do not. IL‐6 is a key inflammatory cytokine in AAA formation.^49^ In the murine AngII infusion model, IL‐6 expression was highest in the adventitia and correlated anatomically with macrophage precursor recruitment.^50–51^ Also, expression of IL‐6 was markedly increased in human AAA compared with atherosclerotic, nonaneurysmal aorta.^12,29^ Further, we observed that CD14 expression was much higher in human AAA and colocalized with macrophages and that monocyte binding to AngII‐infused aorta was CD14 dependent. Collectively, these findings suggest that IL‐6 and CD14 participate in a proinflammatory feed‐forward loop that promotes adventitial inflammation in AAA.

In summary, we report that the innate immune signaling molecule CD14 plays a key role in the pathogenesis of AAA. Expression of CD14 mediates macrophage precursor recruitment to the aortic adventitia in response to soluble factors such as IL‐6 released by fibroblasts and perivascular adipocytes. Expression of CD14 is both locally and systemically upregulated in human AAA. Thus, targeting CD14‐dependent inflammatory responses could represent a fruitful approach to treating AAA in humans.
